# Investigation of Failure Mechanisms in Ceramic Composites as Potential Railway Brake Disc Materials

**DOI:** 10.3390/ma13225141

**Published:** 2020-11-15

**Authors:** Jeongguk Kim

**Affiliations:** Advanced Railroad Vehicle Division, Korea Railroad Research Institute, Uiwang 16105, Korea; jkim@krri.re.kr; Tel.: +82-31-460-5518

**Keywords:** ceramic composites, mechanical properties, tensile tests, microstructural analysis, failure mode, failure mechanism

## Abstract

Ceramic composite materials have been efficiently used for high-temperature structural applications with improved toughness by complementing the shortcomings of monolithic ceramics. In this study, the fracture characteristics and fracture mechanisms of ceramic composite materials were studied. The ceramic composite material used in this study is Nicalon ceramic fiber reinforced ceramic matrix composites. The tensile failure behavior of two types of ceramic composites with different microstructures, namely, plain-weave and cross-ply composites, was studied. Tensile tests were performed on two types of ceramic composite material specimens. Microstructure analysis using SEM was performed to find out the relationship between tensile fracture characteristics and microstructure. It was found that there was a difference in the fracture mechanism according to the characteristics of each microstructure. In this study, the results of tensile tests, failure modes, failure characteristics, and failure mechanisms were analyzed in detail for two fabric structures, namely, plain-weave and cross-ply structures, which are representative of ceramic matrix composites. In order to help understanding of the fracture process and mechanism, the fracture initiation, crack propagation, and fracture mechanism of each composite material are schematically expressed in a two-dimensional figure. Through these results, it is intended to provide useful information for the design of ceramic composite materials based on the mechanistic understanding of the fracture process of ceramic composite materials.

## 1. Introduction

Ceramic composites are structural materials used at high temperatures that have been proven over the past few decades [1,2,3,4]. For this reason, it has been spotlighted as an excellent material in spacecraft insulation materials, high-temperature gas turbine rotors, and thermal management systems, and, recently, it is expanding its use as a material for brake discs for automobiles and railways [1,2,3,4,5,6]. Stadler et al. studied ceramic composite materials as friction materials [7]. In recent years, it has also been used as a braking friction material in the railroad field, increasing its utilization [6,7,8,9]. Stadler et al. proposed carbon fiber reinforced SiC composites as a railway friction material, and reported that the properties of this material show excellent corrosion and oxidation resistance, and have excellent friction and wear characteristics. It was also recommended that ceramic composites are the material that can be protected from the high temperatures occurring on the disc surface during braking [7]. In general, monolithic ceramics exhibit excellent high-temperature properties, corrosion resistance, abrasion resistance, etc., but their use is limited due to the essential characteristics of brittleness. Therefore, the development of a technology that imparts toughness to such monolithic ceramics has become important, and one solution to this is ceramic composite materials reinforced with ceramic fibers [1,2,3,4,5,10,11].

**Figure 1:**
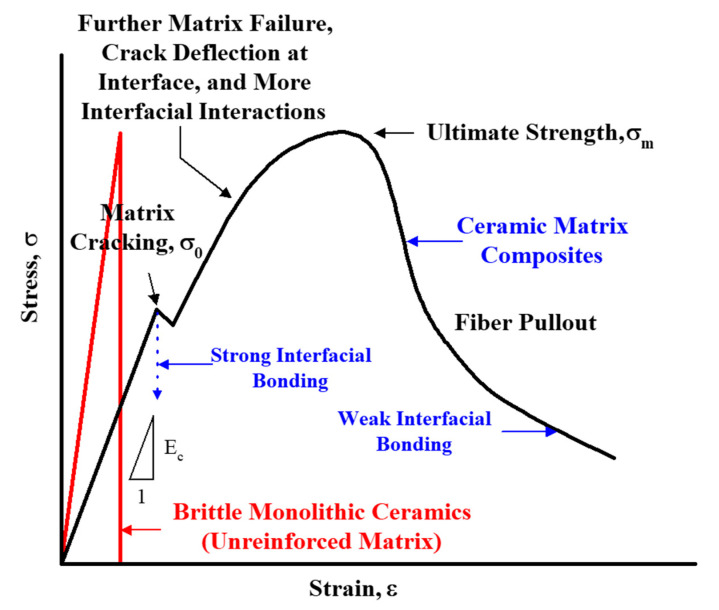
A schematic comparison of fracture behavior between monolithic ceramics and ceramic composites [3].

Figure 1 schematically illustrates a comparison of the fracture behavior between monolithic ceramics and ceramic composites. As shown in Figure 1, monolithic ceramics show a typical brittle fracture behavior that leads to failure immediately after undergoing elastic deformation without a plastic deformation region. On the other hand, in the case of the ceramic composite material reinforced with continuous fibers, it initially shows the elastic behavior of monolithic ceramics, and then shows the first matrix cracking as the load applied to the specimen increases. However, as the stress increases, the continuous increase in load continues due to complex interactions such as further matrix failure due to fibers present inside the matrix and crack deflection at the interface between the fibers and the matrix (Figure 1) [3,4,10,11,12]. After the ultimate tensile stress (UTS) is reached, the composite shows a stress-strain behavior like the shape of plastic deformation exhibited by a metallic material due to the sequential pullout of the fibers until the final failure. Due to this fracture mechanism, it is possible to improve the fracture toughness of monolithic ceramics. In other words, the fracture toughness increases by the larger area in the stress-strain curve. In addition, Figure 1 shows that interfacial bonding between fiber and matrix material is also an important factor. If there is a strong interfacial bonding between the fiber and the matrix, the fiber reinforcement effect will not be seen, and if there is proper interfacial bonding between the fiber and matrix, a ceramic composite material with reinforced toughness as shown in Figure 1 can be expected.

**Figure 2:**
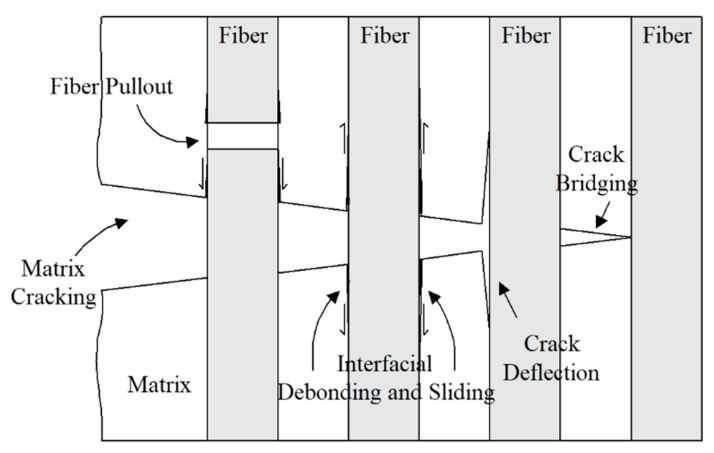
A schematic representation of typical failure patterns of ceramic composites.

Figure 2 schematically shows the typical failure patterns found in ceramic composites [3,4,10]. As the load increases after the initial matrix cracking, phenomena such as interfacial debonding and sliding, crack deflection, and crack bridging between the fiber and the matrix material go through. After that, the fiber is finally broken, and the final failure mechanism is completed through extensive fiber pullout. The fracture toughness of the ceramic composite material is improved by delaying the final fracture through those energy absorption mechanisms [10]. Marshal et al. investigated the failure mechanisms in ceramic matrix composites [10].

**Figure 3:**
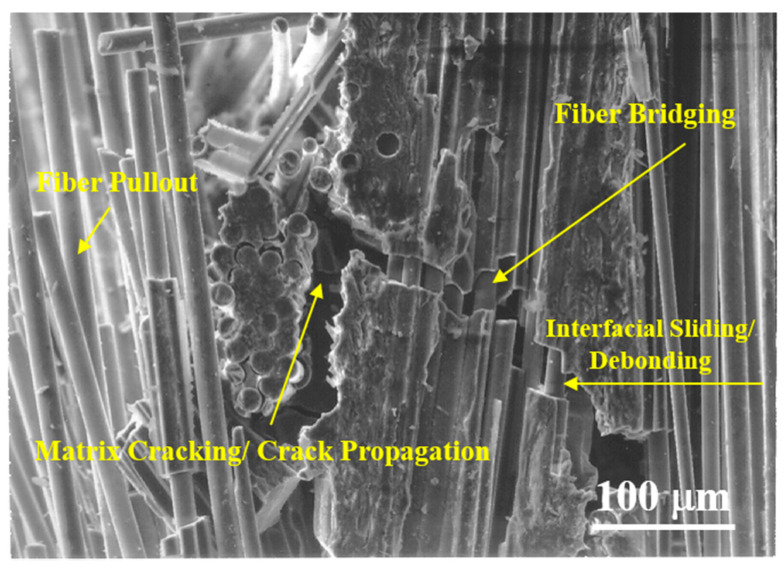
Typical examples of failure mechanisms in ceramic matrix composites as failure energy absorption mechanism.

Figure 3 shows an example of the schematic representation shown in Figure 2. It is obtained from the fracture surface of the actual ceramic composite material, and it shows in detail the process of absorbing fracture energy such as matrix cracking and propagation of crack, interfacial sliding and debonding between fiber and matrix, fiber bridging, and fiber pullout [10,13,14].

Until now, there have been many studies on the mechanical properties and failure of ceramic composites, but relatively few studies on the fracture mechanism and detailed fracture process have been carried out [10,14,15,16,17,18]. Therefore, in this study, as discussed above, the fracture behavior of the actual ceramic composite material was studied, and a study was conducted on the fracture mechanism that can support the fracture behavior. After tensile testing of ceramic composite materials, a study on the failure path and failure mechanism was conducted through the analysis of the specimen using an electron microscope. Based on this research, it is the main objective of this study to provide schematic explanations of the failure mode and mechanism after analysis of the process of fracture. Therefore, the main objectives of this investigation are to (1) investigate the fracture mechanisms of Nicalon/SiC composites both plain-weave and cross-ply composites, (2) conduct microstrucural analyses in tensile failured specimens, (3) visualize the failure mechanisms with two-dimensional schematic presentation, and (4) provide mechanistic understanding of failure characteristics of Nicalon/SiC composites for the design of ceramic matrix composites.

## 2. Materials and Experimental Procedures

The ceramic composite material used in this study is Nicalon ceramic fiber reinforced SiC ceramic matrix composite (Nicalon/SiC). Nicalon/SiC composites are representative ceramic composites that are used in various applications such as ceramic rotors and heat exchangers, etc. [1,2,3,4]. These composites can be used as friction materials in the future railway field, therefore, they were applied in this study. Nicalon fiber is a silicon carbide-based ceramic fiber with a diameter of about 10 microns, and the Nicalon/SiC composite was fabricated using the isothermal chemical vapor infiltration (ICVI) method.

Table 1 shows the mechanical properties data of Nicalon fiber and SiC. In this study, two types of specimens were used, plain-weave Nicalon/SiC composite and 0/90 cross-ply Nicalon/SiC composite. The fiber volume content was 40 wt% for both types of composite materials. 

**Figure 4:**
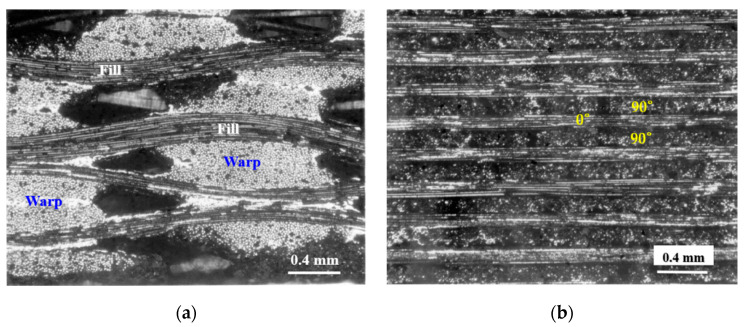
The microstructures of (**a**) plain-weave and (**b**) cross-ply Nicalon/SiC composites.

**Figure 5:**
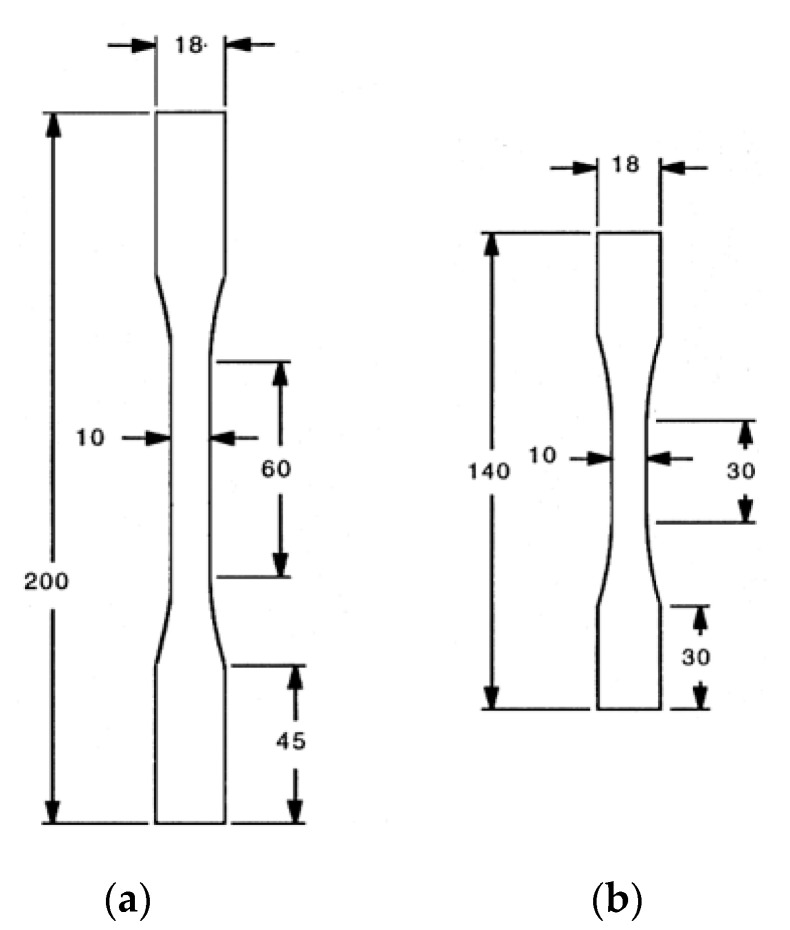
The geometries of tensile test specimens. All dimensions in mm. The Radius of curvature in the shoulder section: 100 mm. (**a**) plain-weave specimen. (**b**) cross-ply specimen.

**Figure 6:**
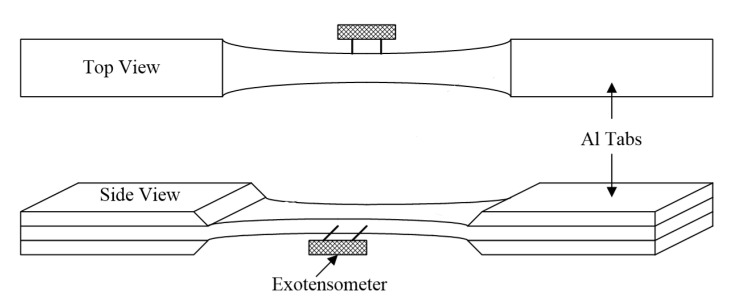
A schematic illustration of mechanical testing setup with specimen.

Figure 4 provides a cross-sectional views of the two composite materials. Figure 5 shows the size and geometry of the specimen, and the thickness of the specimen is about 3 mm. Monotonic tensile test was carried out at room temperature using MTS testing equipment (MTS 810, MTS, Eden Prairie, MN, USA). The tensile test was performed under displacement control with a cross-head speed of 0.3 mm/min. The strain was measured by attaching an axial extensometer (MTS, Eden Prairie, MN, USA) to the grip part of the specimen (Figure 6), and the tensile test was performed according to the ASTM guidelines [19]. Since the specimen used in this study is a ceramic-based specimen, an aluminum tab was attached to the grip portion to prevent the grip portion from being distorted when the specimen was mounted on the tensile tester as shown in Figure 6. For the tensile tests, three samples were prepared and tested for each composite material. After the tensile test, microstructural analysis was performed using SEM (S-360, Cambridge Instruments, Cambridge, UK). After that, in order to provide information to readers in an easy way, the result of this analysis was visualized as a two-dimensional figure to express the final failure process.

## 3. Results and Discussion

**Figure 7:**
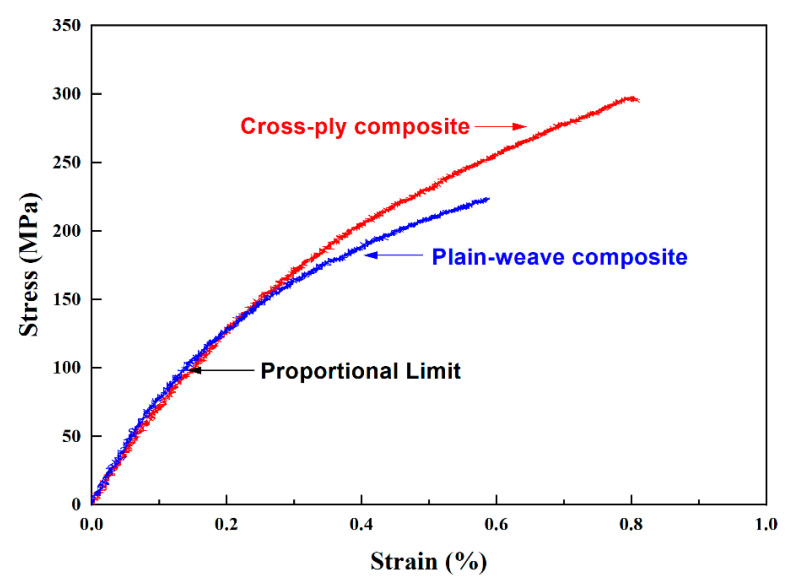
The result of tensile test for Nicalon/SiC ceramic composite.

Figure 7 shows the tensile test results of the Nicalon/SiC ceramic composite material. In common for both specimens, the proportional limit value was obtained around 100 MPa, showing the elastic region up to this point (Figure 1). After that, as the load on the specimen increases, the load curve becomes increasingly nonlinear. Both composite material specimens were finally fractured near the ultimate tensile stress (UTS), and the maximum tensile stresses were about 225 and 300 MPa for plain-weave and cross-ply composites, respectively. The tensile load of the cross-ply composite specimen was found to be higher, which is due to the microstructural influence, and it is believed that the plain-weave composite material is structurally more vulnerable to loads than the cross-ply structure [1,4]. As shown in Figure 1, it can be seen that the failure pattern due to plastic deformation of the metallic material according to fiber pullout after UTS was not shown. This is because, even if the two specimens are finally destroyed by fiber pullout, the energy absorption mechanism varies depending on the stiffness of the fiber or the coating conditions of the fiber. In addition, it is possible to infer that the fiber bundles were destroyed at the same time at the final fracture.

**Figure 8:**
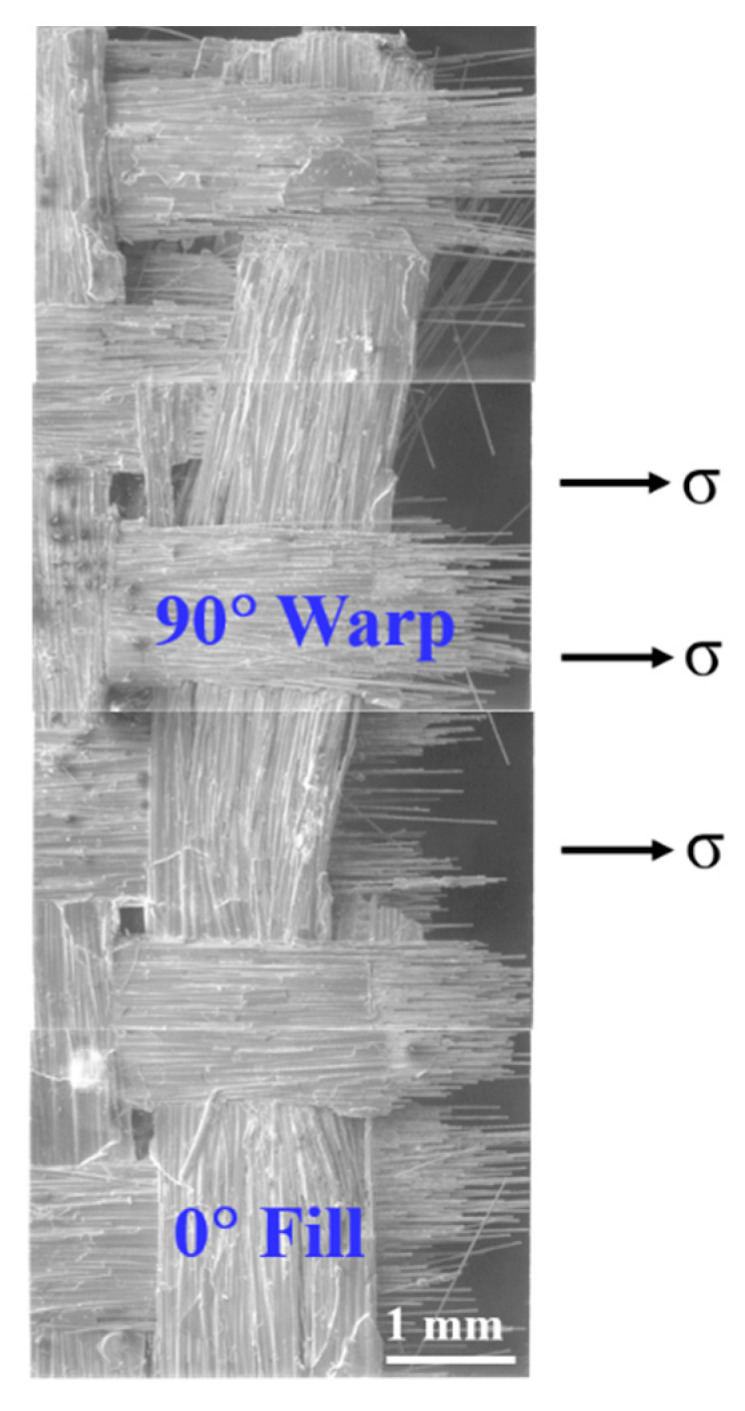
Planar view of fracture surface of plain-weave composite.

**Figure 9:**
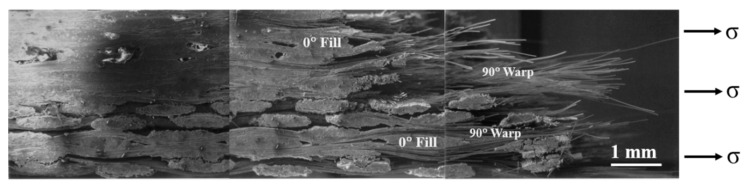
Cross-sectional view of fracture surface of plain-weave composite.

Figure 8 shows the fracture surface in a plain-weave composite. Figure 9 shows a cross-sectional view of the fracture surface of a plain-weave Nicalon/SiC composite. As shown in Figure 8, the 90° warps are oriented in the direction of stress application, so it can be understood that the specimen was finally ruptured through the final fiber fracture. On the other hand, in the case of 0° fills, since they are woven horizontally with 90° warps, the crack propagates at 90° warps after the first crack, and plays a role in giving time so that the final failure can occur at 90° warps. This can be inferred through the cross-sectional view in Figure 9. As shown in Figure 8 and Figure 9, the final failure occurred at 90° warp in the same orientation as the stress applied direction, and it can be seen that the 0° fill plays a role in supporting the final load for failure (Figure 8). Figure 9 also shows a fracture surface similar to that shown in Figure 8. However, when viewed through the cross-sectional view, the role of the 0° fill and 90° warp in the fracture process can be clearly understood [1,2,4].

**Figure 10:**
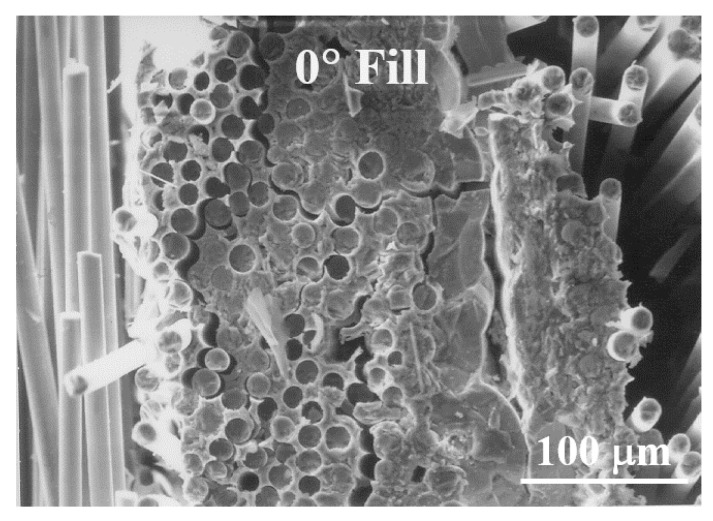
Multiple matrix cracking of 0° fill in plain-weave composite.

**Figure 11:**
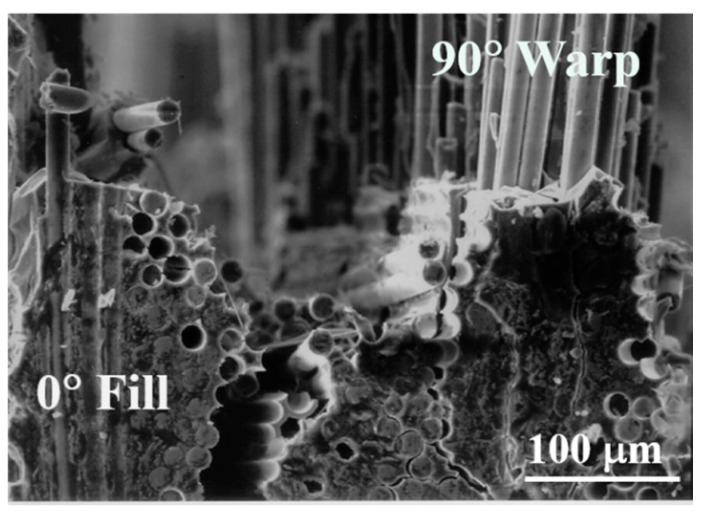
Fiber pullout of the 90° warp followed by severe matrix cracking of the 0° fill in plain-weave composite.

**Figure 12:**
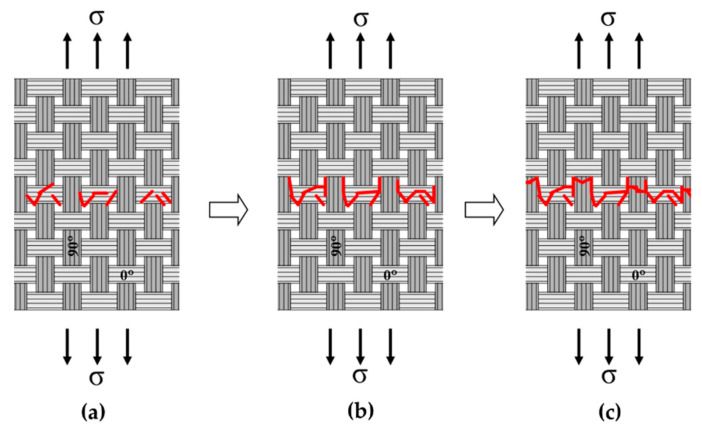
Failure mechanisms in plain-weave Nicalon/SiC composite; (**a**) matrix cracking in 0° fills and crack propagation within 0° fills, and debonding between fiber and matrix in 0° fills, (**b**) delamination in 0° fills and 90° warps and further crack propagation, and debonding between fiber and matrix in 90° warps, (**c**) fiber fracture and extensive fiber pullout in 90° warps.

Figure 10 and Figure 11 show more detailed fracture surfaces. In Figure 10, the 0° fill shows the crack in the SiC matrix. The matrix crack can be seen, which is the starting point of failure caused by the initial load (Figure 10). After that, the crack continues to propagate at the 0° fill (Figure 10). Subsequently, debonding occurs between the fiber and the matrix, and according to the continuously applied load, it reaches 90° warp. Through this fracture process, it can be seen that the fiber pullout at 90° warp and finally fiber breakage lead to final rupture. This process is illustrated schematically, which is described in detail in Figure 12 [4]. Through this process, it is possible to explain the fracture process of the plain-weave Nicalon/SiC composite material.

**Figure 13:**
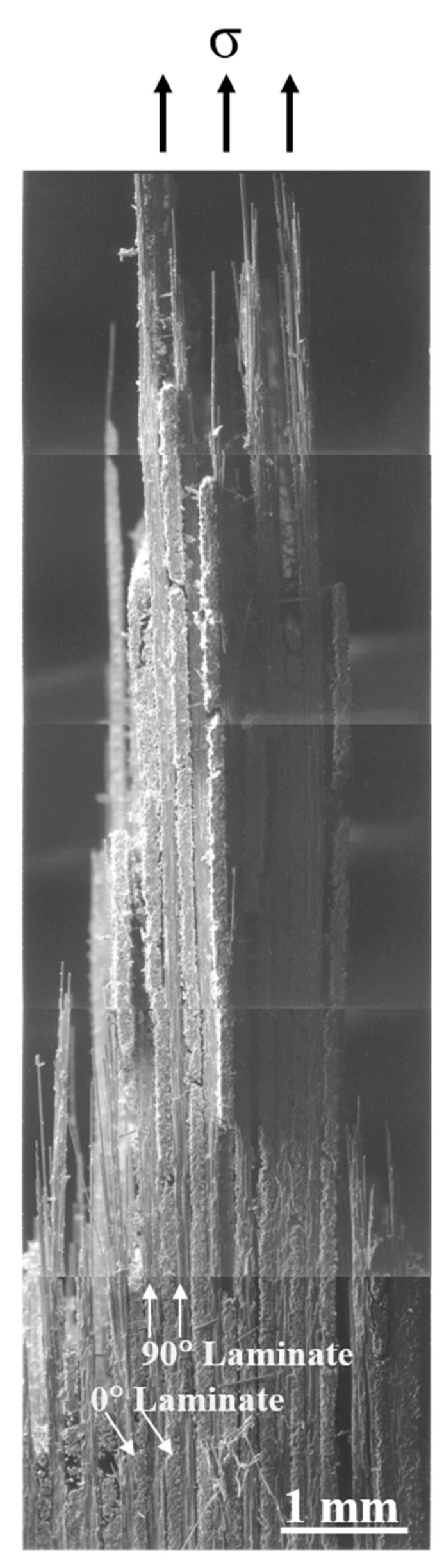
Cross-sectional view of fracture surface of cross-ply composite.

**Figure 14:**
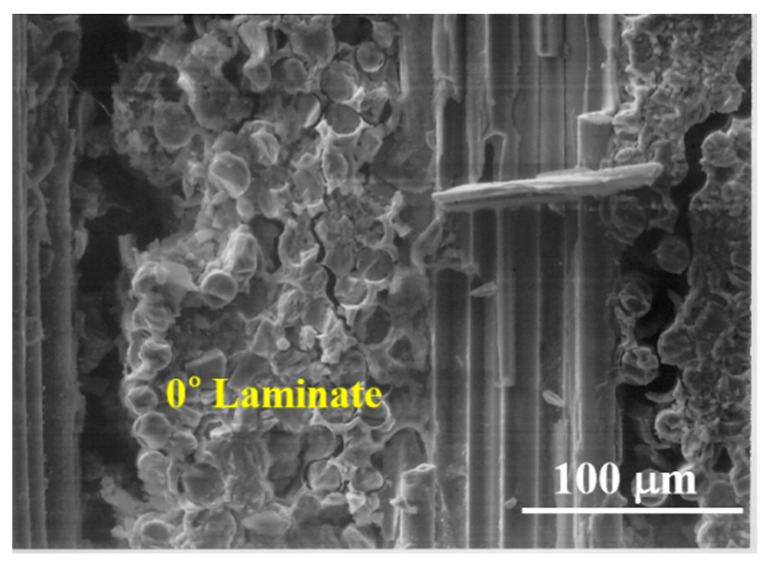
Delamination between fiber and matrix in 0° laminate.

**Figure 15:**
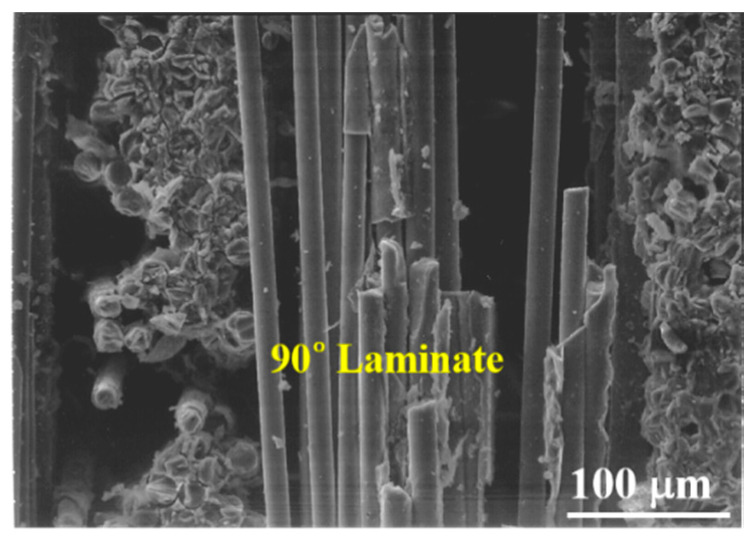
Final failure of the 90° laminate with fiber pullout.

**Figure 16:**
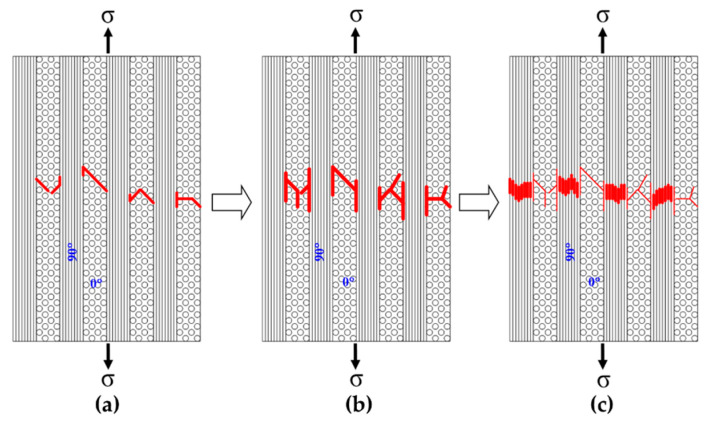
Failure mechanisms in cross-ply Nicalon/SiC composite; (**a**) matrix cracking in 0° laminate and debonding between fiber and matrix at the 0° laminate, (**b**) further crack propagation in 0° laminate and delamination between 0° and 90° laminates, (**c**) fiber debonding and pullout in 90° laminate and final rupture with extensive fiber pullout.

Figure 13 shows the fracture surface of a cross-ply Nicalon/SiC composite. It can be seen that the 0° and 90° laminates are intersected and the final failure occurred through the 90° laminates arranged parallel to the stress applied direction (Figure 13). Figure 14 and Figure 15 show more detailed fracture surfaces. As shown in Figure 14, as a load is applied, the matrix cracking occurs preferentially in the 0° laminate (Figure 14), and then debonding between the matrix and fibers occurs, and crack propagation occurs in the 90° laminate direction. It can be inferred that the final breakdown consists of massive fiber pullout and a final fiber breakage in the 90° laminate (Figure 15). Similar to the description of the failure process of plain-weave composites, the failure of cross-ply composites is schematically described in Figure 16. In other words, first matrix cracking starts in the 0° laminate, followed by debonding between the fiber and the matrix in the 0° laminate. After that, crack propagation continues in the 0° laminate. Thus, as the load increases, delamination occurs between the 0° and 90° laminates. Subsequently, fiber debonding and pullout are performed on a 90° laminate. The final fracture is completed with massive fiber pullout and fiber breakage in a 90° laminate [1,3].

## 4. Conclusions

In this study, tensile fracture characteristics of Nicalon ceramic fiber reinforced ceramic matrix composites were studied. It was found that the composite materials of plain-weave and cross-ply structures showed different tensile fracture characteristics. It was found that the cross-ply composite material showed higher tensile strength than the plain-weave composite, which seems to be due to the microstructural characteristics. The failure mode and failure characteristics of each composite system were found through SEM microstructure analysis, and the failure mechanisms were summarized schematically.

In the case of plain-weave composite material, it was found that the crack of the silicon carbide matrix started at 0° fill. After that, the crack continued to propagate at 0° fill, and then debonding between the fiber and the matrix occurred, and the crack reached 90° warp according to the continuously applied load. Through this fracture process, it was found that the fiber pullout at 90° warp and finally fiber breakage lead to final rupture.

In the case of cross-ply composites, first matrix cracking begins in the 0° laminate, followed by debonding between the fiber and the matrix in the 0° laminate. After that, crack propagation occurs continuously in the 0° laminate. Thus, as the load increases, delamination occurs between the 0° and 90° laminates. Subsequently, fiber debonding and pullout are performed on a 90° laminate. It can be seen that the final rupture completes the failure process by massive fiber pullout and fiber breakage.

## Figures and Tables

**Table 1:** The mechanical properties of Nicalon fiber and SiC [1].

Materials	Tensile Strength (GPa)	Densigy (g/cm^3^)
Nicalon	200	2.5
SiC	240	3.21

## References

[1] Chawla K.K. (2012). Composite Materials: Science and Engineering.

[2] Krenkel W. (2008). Ceramic Matrix Composites: Fiber Reinforced Ceramics and their Applications.

[3] Kim J. (2019). Tensile Fracture Behavior and Characterization of Ceramic Matrix Composites. Materials.

[4] Kim J., Liaw P.K. (2007). Tensile fracture behavior of Nicalon/SiC composites. Met. Matls Trans. A.

[5] Krupka M., Kienzle A. (2000). Fiber Reinforced Ceramic Composite for Brake Discs. Proceedings of the 18th Annual Brake Colloquium & Engineering Display.

[6] Li W., Yang X., Wang S., Xiao J., Hou Q. (2020). Comprehensive Analysis on the Performance and Material of Automobile Brake Discs. Metals.

[7] Stadler Z., Krnel K., Koamac T. (2008). Friction and wear of sintered metallic brake linings on a C/C-SiC composite brake disc. Wear.

[8] Bharambe A. (2016). Medelling and Analysis of Disc Brake with Composite Material. Int. J. Sci. Res..

[9] Benseddiq N., Weichet D., Seidermann J., Minet M. (1996). Optimization of design of railway disc brake pads. J. Rail. Rapid Transit..

[10] Marshall D.B., Evans A.G. (1985). Failure mechanisms in ceramic-fiber/ceramic-matrix-composites. J. Am. Ceram. Soc..

[11] Pickering K.L., Efendy M.A., Le T.M. (2016). A review of recent developments in natural fibre composites and their mechanical performance. Compos. A Appl. Sci. Manuf..

[12] Tracy J.M. (2014). Multi-Scale Investigation of Damage Mechanisms in SiC/SiC Ceramic Matrix Composites. Ph.D. Thesis.

[13] Ding Z., Li Y., Lu C., Liu J. (2018). An investigation of fiber reinforced chemically bonded phosphate ceramic composites at room temperature. Materials.

[14] Jenkins M.G., Singh R.N. (2001). Failure analysis of ceramic-matrix composites. ASM Handbook. Vol. 21. Composites.

[15] Dowling N.E. (2012). Mechanical Behavior of Materials.

[16] Morscher G.N. (1999). Modal acoustic emission of damage accumulation in a woven SiC/SiC composite. Comput. Sci. Technol..

[17] Kim J., Liaw P.K. (2005). Characterization of fatigue damage modes in Nicalon/Calcium aluminosilicate composites. J. Eng. Mater. Technol..

[18] Kim J., Liaw P.K. (2005). Monitoring tensile damage evolution in Nextel312/Blackglas composites. Mater. Sci. Eng. A.

[19] ASTM (2018). C1275-18 Standard Test Method for Monotonic Tensile Behavior of Continuous Fiber-Reinforced Advanced Ceramics with Solid Rectangular Cross-Section Test Specimens at Ambient Temperature.

